# Safety and Effectiveness of Mechanical Thrombectomy From the Fully Enrolled Multicenter, Prospective CLOUT Registry

**DOI:** 10.1016/j.jscai.2023.100585

**Published:** 2023-02-23

**Authors:** David Dexter, Herman Kado, Abdullah Shaikh, Jonathan Schor, Suman Annambhotla, Adam Zybulewski, Joseph Paulisin, Mohannad Bisharat, Nicolas J. Mouawad, Matthew C. Bunte, Thomas Maldonado, Edvard Skripochnik, Adam Raskin, Sagar Gandhi, Eugene Ichinose, Robert Beasley, Hamid Mojibian

**Affiliations:** a Sentara Vascular Specialists, Norfolk, Virginia; b Ascension Providence Hospital, Farmington Hills, Michigan; c William Beaumont Hospital, Royal Oak, Michigan; d Allegheny Health Network, Pittsburgh, Pennsylvania; e Northwell Health, Staten Island University Hospital, Staten Island, New York; f Longstreet Clinic, Gainesville, Georgia; g Mount Sinai Medical Center of Florida, Miami, Florida; h Ascension Michigan Genesys Hospital, Grand Blanc, Michigan; i Ashchi Heart and Vascular Center, Jacksonville, Florida; j McLaren Bay Heart and Vascular, Bay City, Michigan; k Saint Luke’s Mid America Heart Institute, Kansas City, Missouri; l New York University Langone Health, New York, New York; m Columbia University Irving Medical Center, New York, New York; n Mercy Heart Institute, Cincinnati, Ohio; o Prisma Health, University of South Carolina – School of Medicine, Greenville, South Carolina; p Oklahoma Heart Institute, Tulsa, Oklahoma; q Palm Vascular Centers, Miami Beach, Florida; r Yale School of Medicine, New Haven, Connecticut

**Keywords:** deep vein thrombosis, mechanical thrombectomy

## Abstract

**Background:**

We report in-hospital outcomes from the multicenter, prospective, single-arm ClotTriever Outcomes (CLOUT) registry, which enrolled up to 500 patients with proximal lower extremity deep vein thrombosis (DVT) treated with percutaneous mechanical thrombectomy using the ClotTriever System (Inari Medical).

**Methods:**

The CLOUT registry enrolled all-comer patients with DVT, irrespective of symptom duration, thrombus age, prior treatment of the current DVT, or bilateral thrombus. The primary effectiveness end point was defined as complete or near complete (≥75%) reduction in Marder score. Thrombus burden was assessed by an independent core laboratory. Mortality and serious adverse events, including device-relatedness, were adjudicated by an independent medical monitor. Here, safety and outcomes are evaluated through discharge.

**Results:**

The median age was 61.9 years (IQR, 48.0-70.8), 50.5% were women, 24.9% had a history of DVT, and 23.2% had previously failed treatment of the current DVT. Nearly all procedures (99.4%) were performed in a single session with negligible procedural blood loss (median 40.0 mL; IQR, 20.0-50.0), and most patients (97.8%) required no subsequent intensive care unit monitoring. The primary effectiveness end point was achieved in 91.2% of limbs. Through discharge, 1 device-related serious adverse event (0.2%) occurred. Health status, as assessed by self-reported pain and circumferential measurements of limb edema, were significantly improved at discharge.

**Conclusions:**

Thrombectomy with the ClotTriever System is a safe and effective treatment for proximal lower extremity DVT, while also avoiding the need of intensive care. Early patient improvements are demonstrated, and follow-up is ongoing to 2 years.

## Highlights

• ClotTriever Outcomes is the largest prospective registry evaluating mechanical thrombectomy in deep vein thrombosis.
• 91.2% of limbs had near or complete and 63.8% had complete thrombus removal.
• ClotTriever is safe with a 0.2% device-related serious adverse event rate.
• Patients experienced significant and immediate relief from pain and swelling.

## Central Illustration

**Figure:**
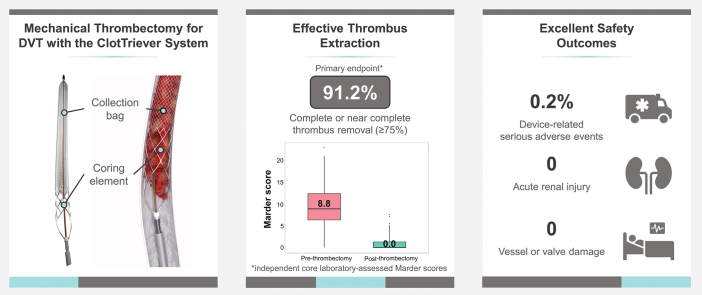

## Introduction

Deep vein thrombosis (DVT) occurs with an annual incidence of 50 to 80 cases per 100,000^1^ and at higher rates among the hospitalized (1.3%),^2^ elderly (1.8%-3.1%),^3^ and those with active malignancy (5.8%-9.6%).^4^ Pulmonary embolism (PE) is a life-threatening complication that develops in up to 25% of patients with DVT. The long-term consequence of DVT can have chronic implications, including diminished quality of life, limitations in daily function, and long-term disability in severe cases, collectively known as postthrombotic syndrome (PTS). Invasive treatment of lower extremity DVT in the proximal veins offers improved quality of life and has the potential of reducing late morbidity, including subsequent PE and PTS.^5^

Anticoagulation is recommended as the front-line therapy for DVT^6^ and can effectively prevent further thrombus formation. However, anticoagulation is inadequate at resolving existing thrombus. Relative to anticoagulation, catheter-directed thrombolysis (CDT) has produced inconclusive benefit.^7^, ^8^, ^9^, ^10^, ^11^, ^12^, ^13^, ^14^ Furthermore, thrombolytic therapies have been shown to be less effective when treating older, chronic thrombus^15^ and carry additional bleeding risk. Consequently, there is growing interest in percutaneous mechanical thrombectomy (MT). Clinical guidelines continue to recommend conventional treatment with anticoagulation alone in most scenarios for DVT because of limited clinical evidence supporting interventions.^16^, ^17^, ^18^

Interventions with MT, using purely mechanical means or in combination with thrombolytics, involve endovascular removal, rather than dissolution of thrombus. In comparison with thrombolytic-based therapies, purely mechanical MT can avoid added bleeding risks and reduce intensive care unit (ICU) monitoring that is requisite with CDT. The ClotTriever System (Inari Medical) is an MT device designed for the nonsurgical removal of venous thrombus. The prospective, multicenter ClotTriever Outcomes (CLOUT) registry was designed to assess the safety and effectiveness of the ClotTriever System for the treatment of proximal, lower extremity DVT in an encompassing, real-world population. Previously, results from an interim patient analysis showed the safety and efficacy of MT.^19^ Herein, the in-hospital outcomes are reported from the fully enrolled CLOUT registry.

## Methods

### Study design and patient population

The CLOUT registry (NCT03575364) is a prospective, single-arm study evaluating outcomes of all-comer patients with proximal lower extremity DVT after thrombectomy with the ClotTriever System. The registry enrolled 500 patients from 43 US clinical sites. The study was conducted in accordance with the ethical principles outlined in the Declaration of Helsinki, Good Clinical Practice principles, and ISO 14155:2011. Institutional review board approval (WCG IRB) was obtained for the study, and all patients provided informed written consent.

The study enrolled patients aged ≥18 years with a lower extremity DVT in unilateral or bilateral iliofemoral veins. Thrombus could extend into the distal inferior vena cava (IVC). Patients were included irrespective of symptom duration, prior treatment of the current DVT, contraindication to thrombolytics, or COVID-19 status. Failed prior treatment was defined as unsuccessful endovascular intervention (mechanical, pharmacologic, or pharmacomechanical) or anticoagulation for at least 1 week. Exclusion criteria included life expectancy <1 year, prior venous stenting of a target vessel segment, IVC atresia or other congenital anomalies of the IVC or iliac veins, prior IVC filter in place, inability to receive anticoagulation or contrast, chronic nonambulatory status, known hypercoagulable state that could not be medically managed, and unavailability of a proximal lower extremity venous access site.

### Study device and procedure

**Figure 1:**
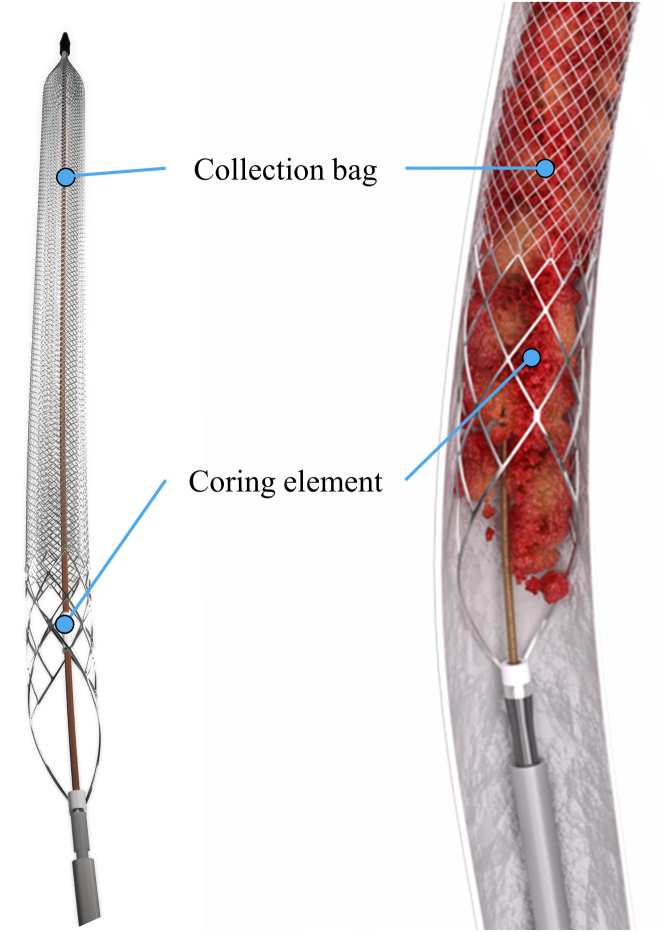
Left: Overview of ClotTriever System components with nitinol coring element and collection bag. Right: Demonstration of the study device deposing wall-adherent thrombus with the coring element before collapsing and removing the collection bag. Images are courtesy of Inari Medical.

The ClotTriever System consists of the following: (1) a proprietary introducer sheath (13F or 16F) with an expandable funnel and (2) a ClotTriever catheter with coring element and collection bag (Figure 1, left). After gaining access, the catheter is advanced overwire through the sheath past the thrombus. The collection bag is then opened, and the catheter is slowly retracted to envelop the thrombus (Figure 1, right). Once retracted to the sheath, the collection bag is collapsed, and the catheter removed. The device can then be cleared of thrombus and reintroduced for additional passes per physician preference.

The study procedure included balloon venoplasty, stenting, or further adjunctive therapy (eg, thrombolytic or other thrombectomy procedure with a different device), per the investigator’s discretion. The postthrombectomy anticoagulation regimen was unconstrained by the study protocol. Intravascular ultrasound was recommended pre- and postprocedure.

### Baseline and procedural characteristics

The CLOUT registry captured demographic and procedural information. Thrombectomy time was measured from the first insertion of the study device catheter to its final removal. Thrombus chronicity was assessed by the treating physician at 3 separate time points: from medical history, diagnostic imaging, and the texture and visual appearance of extracted thrombus postthrombectomy. Thrombus chronicity was defined according to the oldest thrombus present per treated limb and was either acute (<2 weeks), subacute (2-6 weeks), or chronic (>6 weeks).

### Primary effectiveness end point

**Figure 2:**
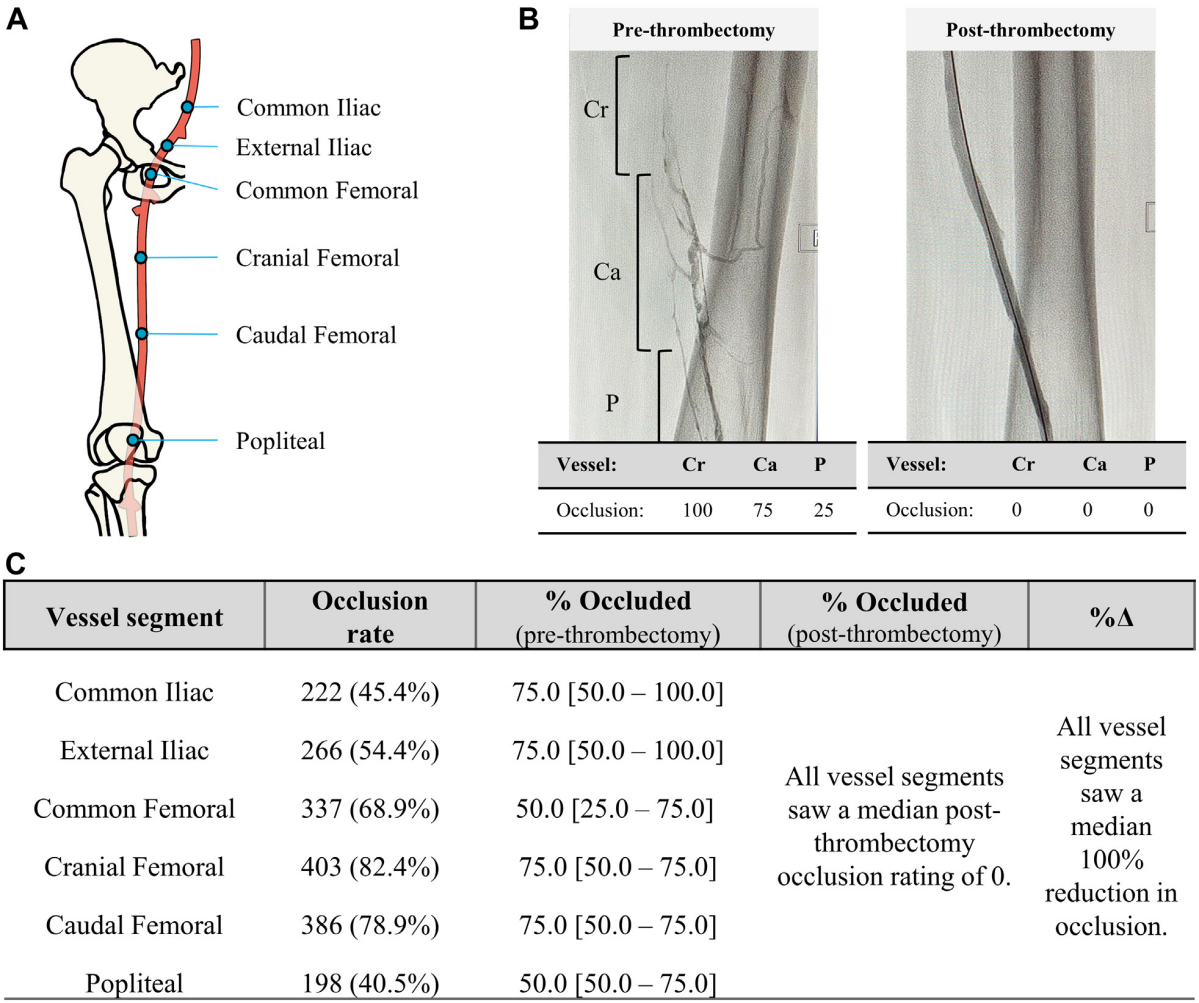
(**A**) Anatomic diagram of the treated vessel segments. (**B**) Example of pre- and postthrombectomy venography and occlusion rates. (**C**) Summary information of occlusion rate and median percent occluded [interquartile range] in each vessel segment. Ca, caudal femoral vein; Cr, cranial femoral vein; P, popliteal vein.

The primary effectiveness end point of the study was complete or near complete (≥75%) reduction in Marder score as a quantitative venous assessment following thrombectomy. Marder scores were assessed by an independent core laboratory (NAMSA). Thrombus burden in each target vessel segment (Figure 2A) was categorically scored as either 0% (no occlusion), 25%, 50%, 75%, or 100% (complete occlusion) (Figure 2B). Scores were then weighted based on vessel size, yielding a maximum Marder score of 24 for proximal DVT. Marder scores were calculated preprocedure and postprocedure.

### Safety and clinical outcomes

Serious adverse events (SAEs) were adjudicated by an independent medical monitor through discharge. SAEs were defined as untoward medical occurrences or exacerbation of preexisting medical conditions that meet at least one of the following criteria: fatal, life-threatening, or resulting in persistent or significant disability, permanent impairment of body function, or permanent damage to a body structure; necessitating or prolonging hospitalization; or necessitating intervention to preclude permanent impairment or damage. Relatedness to the study device or procedure was also assessed.

Acute symptom relief was represented by the change in edema and pain measurements from baseline to discharge. Edema was recorded as the circumference of the treated limb at the midcalf and midthigh. Pain intensity was rated from 0 to 10 and was measured by patient self-assessment using the numeric pain rating scale (NPRS). Hospital and ICU lengths of stay were calculated with 1 day being equal to 1 overnight stay.

### Statistical analysis

Baseline and outcome metrics are summarized using descriptive statistics. Categorical variables are reported as counts (%) and were compared with the generalized McNemar’s test using paired data. Continuous variables are reported as medians and interquartile ranges (IQRs) and were compared with the Wilcoxon signed-rank test using paired data. The percent change of a variable (eg, Marder score) across timepoints was calculated as $\left(\frac{pretreatment-posttreatment}{pretreatment}\right)\times 100$. *P* values of <.05 were considered significant for hypothesis testing. Statistical analyses were performed with SAS 9.4 (SAS Institute Inc) and R 4.0.4 (RStudio Inc).

## Results

### Baseline characteristics

The CLOUT Registry enrolled 500 patients. One patient did not meet the study criteria (life expectancy <1 year) due to preexisting malignancy and was excluded postenrollment. The final analysis population for this study is 499 patients (521 treated limbs).

Baseline characteristics are presented in Table 1. The median age was 61.9 years (IQR, 48.0-70.8), 49.5% were men, 77.0% were White, and 29.7% had a relative or absolute contraindication to thrombolytic therapy. Nearly one-quarter of patients had a prior history of DVT (24.9%) or experienced unsuccessful treatment of the current DVT (23.2%) before enrollment. Although the largest group of patients presented with symptom duration <7 days (48.8%), a comparable amount (48.3%) had symptoms for >7 days, including 6.8% with symptoms for >6 weeks. Thrombus in affected limbs was unilateral in 95.6% of patients and was isolated to the iliac or iliofemoral vessels in 11.0%, isolated to the femoral-popliteal vessels in 21.8%, and involved both the iliofemoral and femoral-popliteal vessels in 67.3% of patients. Thrombus was estimated to be acute in 29.8% of limbs, subacute in 34.4% of limbs, and chronic in 35.8% of limbs. Most patients had a clinical, etiologic, anatomic, and pathophysiologic classification of C3 (72.1%) and a median baseline Villalta score of 9.0 (IQR, 5.0-14.5).

**Table 1:** Baseline characteristics.

Baseline characteristic	
Age, y	61.9 [48.0-70.8]
Male sex	247 (49.5%)
Race	
White	376 (77.0%)
Black	101 (20.7%)
American Indian or Alaskan Native	5 (1.0%)
Asian	3 (0.6%)
Other	5 (1.0%)
BMI, kg/m^2^	30.2 [25.8-35.1]
Symptom duration	
<7 d	252 (48.8%)
7-14 d	121 (23.4%)
2-4 wk	70 (13.6%)
4-6 wk	23 (4.5%)
>6 wk	35 (6.8%)
Prior history of DVT	124 (24.9%)
Prior treatment of current DVT	120 (23.2%)
Contraindication to thrombolytic drug therapy	148 (29.7%)
Provoked	206 (40.1%)
Surgery	86 (41.7%)
Immobilization	36 (17.5%)
Childbirth/pregnancy	5 (2.4%)
Unilateral	477 (95.6%)
Left	352 (70.5%)
Right	169 (33.9%)
Bilateral	22 (4.4%)
DVT location	
Isolated iliofemoral	55 (11.0%)
Iliofemoral and femoral-popliteal	337 (67.3%)
Isolated femoral-popliteal	109 (21.8%)
Thrombus chronicity by leg	
Acute (<2 wk)	153 (29.8%)
Subacute (2-6 wk)	177 (34.4%)
Chronic (>6 wk)	184 (35.8%)
Villalta score	9.0 (5.0-14.5)
0-4	82 (18.7%)
5-9	151 (34.3%)
10-14	97 (22.1%)
≥15	110 (25.0%)
CEAP score of treated vessels	
C0-C2	35 (7.4%)
C3	343 (72.1%)
C4-C6	98 (20.6%)
rVCSS	6.0 [3.0-9.0]
EQ-5D	0.69 [0.46-0.83]

Values are presented as n (%) or median [interquartile range]. N varies from 430 to 499 patients or 476 to 518 treated limbs. BMI, body mass index; CEAP, clinical etiological anatomic pathophysiologic; DVT, deep vein thrombosis; EQ-5D, EuroQol group 5-dimension self-report questionnaire; rVCSS, revised venous clinical severity.

### Procedural characteristics

Procedural characteristics are presented in Table 2. All but 3 interventions (99.4%) were completed in a single session, with a median of 4.0 (IQR, 3.0-6.0) passes with the MT catheter and a median thrombectomy time of 26.0 minutes (IQR, 18.0-40.0). Median estimated blood loss was 40.0 mL (IQR, 20.0-50.0) and 3 (0.6%) patients required transfusion. Adjunctive venoplasty was performed in 72.7% of treated limbs, and stents were placed in 44.3%. The rates of adjunctive thrombolytic therapy (0.4%) and other thrombectomy (0.6%) were low. The median postthrombectomy hospital length of stay was 1.0 day (IQR, 1.0-2.0), and only 11 (2.2%) patients were referred for overnight ICU monitoring following the procedure.

**Table 2:** Procedural characteristics.

Procedural characteristic	
Single session	496 (99.4%)
Procedure time, min	65.0 [46.0-87.0]
Thrombectomy time, min	26.0 [18.0-40.0]
Fluoroscopy time, min	13.4 [8.8-20.6]
Number of device passes	4.0 [3.0-6.0]
Blood loss	
Estimated blood loss, mL	40.0 [20.0-50.0]
Patients requiring transfusion	3 (0.6%)
Transfusion volume, units	1.0 [0.75-4.0]
Adjunctive therapies	
Venoplasty	379 (72.7%)
Stenting	231 (44.3%)
Catheter-directed thrombolysis	2 (0.4%)
Percutaneous mechanical thrombectomy	3 (0.6%)
Other	8 (1.5%)
Stay^a^	
Total hospital length of stay, d	2.0 [1.0-4.0]
Postthrombectomy hospital length of stay, d	1.0 [1.0-2.0]
Patients requiring postthrombectomy ICU stay	11 (2.2%)

Values are presented as n (%) or median (interquartile range). N varies from 446 to 499 patients or 507 to 521 treated limbs. ICU, intensive care unit.

a 1 day = 1 overnight stay.

### Thrombus by vessel segment

The most commonly thrombosed vessel segments (Figure 2C) included the common femoral (68.9%), cranial femoral (82.4%), and caudal femoral (78.9%), whereas the least commonly thrombosed vessel was the popliteal vein (40.5%). Most thrombosed vessel segments had a median prethrombectomy occlusion rate of 75%, whereas the common femoral and popliteal veins had median prethrombectomy occlusion rates of 50%. All treated vessel segments saw a significant reduction in thrombus burden and a median thrombus reduction of 100% following thrombectomy (all *P* < .0001) (Figure 2C).

### Effectiveness outcomes

**Figure 3:**
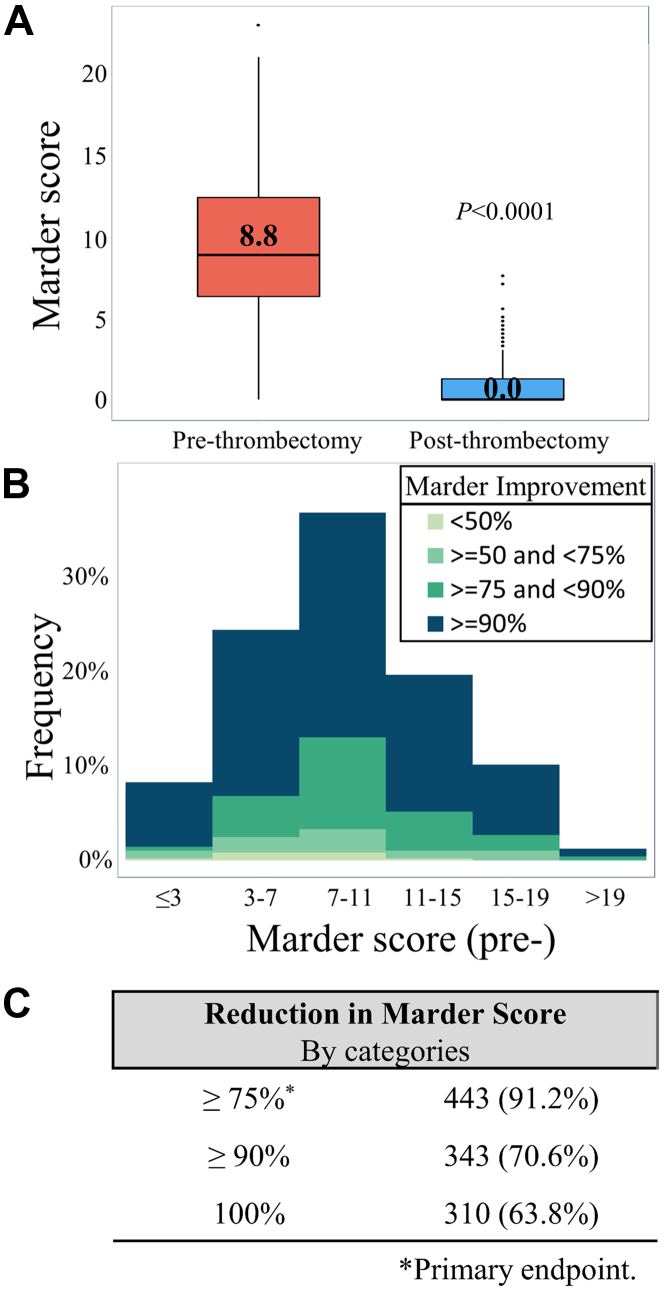
(**A**) Box-and-whisker plots presenting the pre- and postthrombectomy Marder scores. Boxes represent interquartile range (Q1, Q3) with horizontal bars representing median values. Whiskers represent 1.5 × (Q1–Q3). Dots represent outliers beyond the whiskers. (**B**) Histogram of prethrombectomy Marder scores with colors representing categories of percentage Marder score improvement postthrombectomy. (**C**) Summary information of Marder score reduction by category.

The median Marder score improved from 8.75 (IQR, 6.25-12.25) at baseline to 0.0 (IQR, 0-1.25) postthrombectomy (Figure 3A) (*P* < .0001). The primary effectiveness end point of complete or near complete reduction in Marder score by ≥75% was achieved among 91.2% of evaluable limbs, with 70.6% reaching ≥90% thrombus removal (Figure 3B, C) and 63.9% achieving 100% thrombus removal. The change in pre- to postthrombectomy Marder scores are represented in Figure 3B and C.

### Safety outcomes

There were 13 (2.6%) SAEs (Table 3); a single SAE (0.2%) was adjudicated to be device related. The device-related event resulted from embolization of IVC thrombus after the catheter entangled with a separate device, leading to a fatal PE. Of the 13 SAEs, 5 (1.0%) were rethrombosis events and 3 (0.6%) were PE events. Although not directly assessed, there were no reported SAEs of valve or vessel damage or acute kidney injury.

**Table 3:** Safety outcomes through discharge.

Safety outcome	
All-cause mortality	3 (0.6%)
Serious adverse events	13 (2.6%)
Serious adverse events by type	
Rethrombosis or residual thrombus	5 (1.0%)
Pulmonary embolism	3 (0.6%)
Device-related	1 (0.2%)
Cardiac arrest	1 (0.2%)
Epistaxis	1 (0.2%)
Hemoglobin levels decreased	1 (0.2%)
Pulseless electrical activity	1 (0.2%)
Respiratory failure	1 (0.2%)
Acute kidney injury	0
Vessel or valve damage	0

Values are presented as n (%). N = 499 patients.

All-cause mortality through discharge occurred in 3 (0.6%) patients, 1 (0.2%) of which was the result of the device-related PE event described above. The remaining deaths occurred because of the progression of underlying stage IV non–small cell lung cancer (n = 1) and spinal infection following recent lumbar fusion (n = 1). Three (0.8%) patients received reintervention before hospital discharge, but thrombus removal remained unsuccessful.

### Immediate clinical outcomes

**Figure 4:**
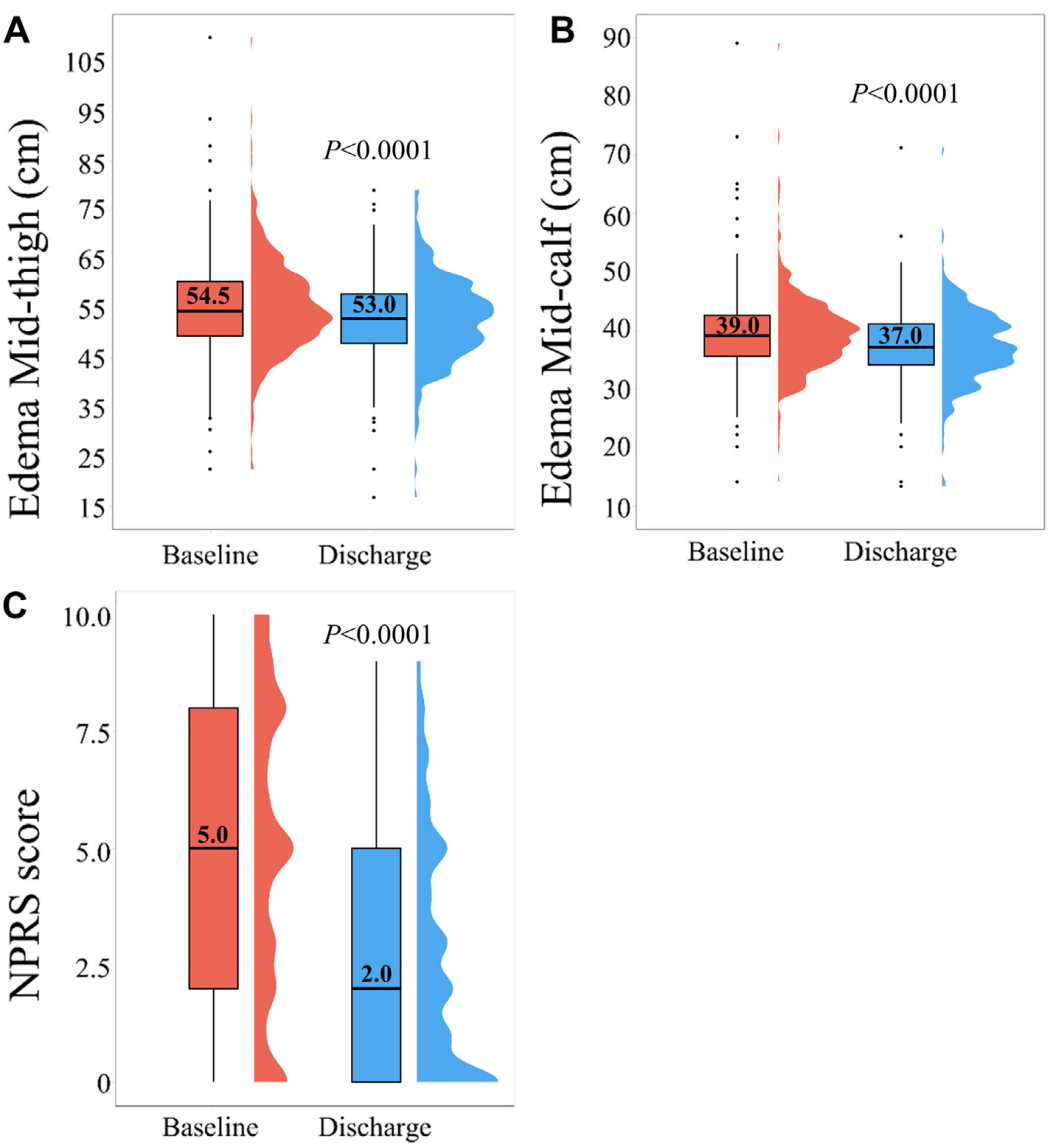
Box-and-whisker with half-violin plots presenting edema pre- and postthrombectomy by (**A**) midthigh and (**B**) midcalf circumferences. (**C**) Box-and-whisker with half-violin plot of pre- and postthrombectomy NPRS score. NPRS, numeric pain rating scale.

Edema and pain significantly improved at discharge (Figure 4). Midthigh circumference was 54.5 cm (IQR, 49.5-60.5) at baseline and reduced to 53.0 cm (IQR, 47.4-58.0) at discharge (*P* < .0001). Midcalf circumference was 39.0 cm (IQR, 35.5-42.5) at baseline and reduced to 37.0 cm (IQR, 34.0-41.0) at discharge (*P* < .0001). Median NPRS scores improved from 5.0 (IQR, 2.0-8.0) at baseline to 2.0 (IQR, 0-5.0; *P* < .0001). Of patients with pain at baseline (eg, NPRS > 0), 76.9% experienced immediate relief (≥1-point NPRS score reduction).

## Discussion

This analysis of in-hospital results from the CLOUT registry demonstrates safe and effective thrombus removal with immediate symptom relief when using the ClotTriever System for treating proximal lower extremity DVT (Central Illustration). These results are impactful as the registry offers liberal inclusion criteria and few exclusions, providing a pragmatic view of MT. In comparison, prior studies have been limited by symptom duration and often exclude patients with high bleeding risk.^7^, ^8^, ^9^, ^10^, ^11^, ^12^, ^13^, ^14^ Therefore, the CLOUT registry offers a substantial interventional data set in patients with DVT that have frequently been excluded in other studies.

**Central Illustration:**
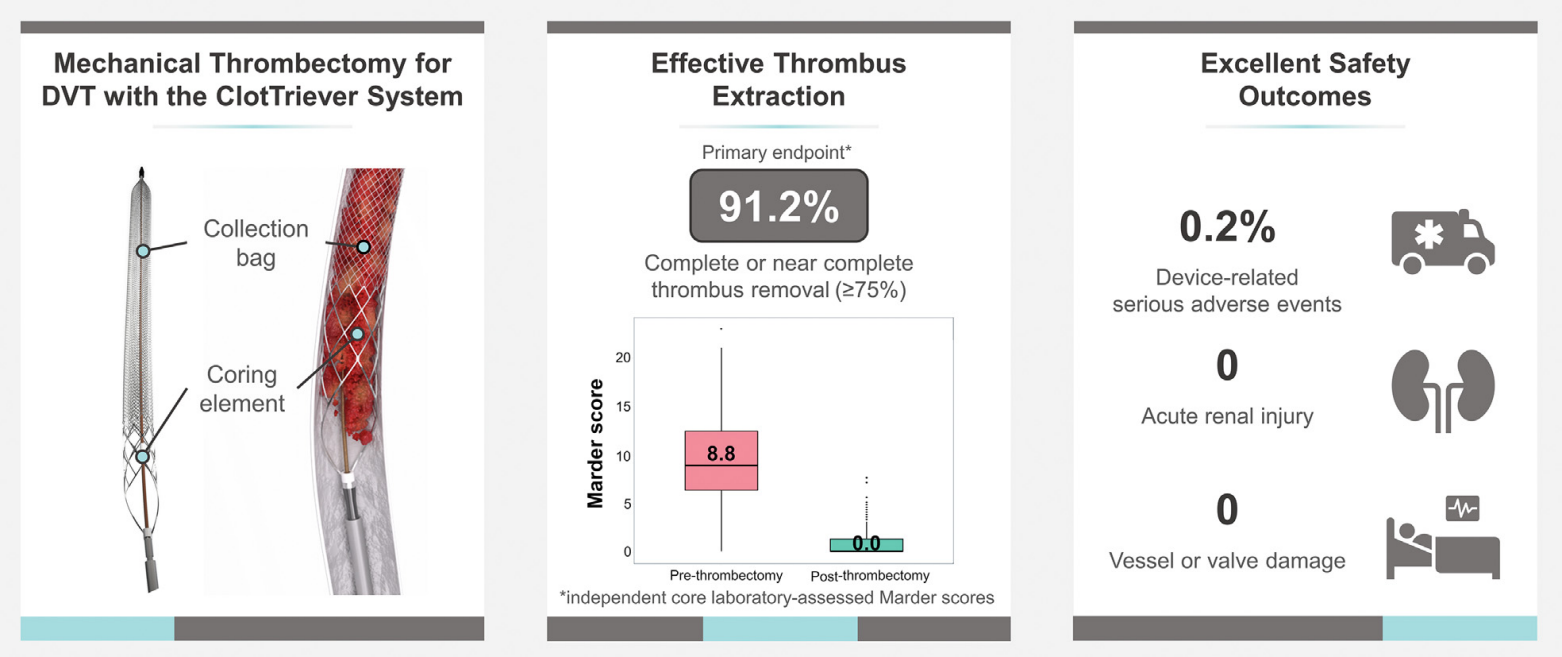
(Left) System overview of the study device. (Middle) Summary of the primary endpoint results. (Right) Key safety outcomes through discharge.

Residual venous thrombus has been reported to occur in 36% to 55% of patients with DVT^20^, ^21^, ^22^ and is an important predictor of PTS,^22^^,^^23^ recurrence,^23^^,^^24^ and mortality.^21^ In this study, core laboratory-assessed venography showed most patients (63.8%) had complete vessel clearance and 91.2% met the primary effectiveness end point. The mean percentage change in Marder score was 92.2%, which is favorable to outcomes from the ATTRACT trial (76.0%).^10^ Analysis at the vessel level (Figure 2C) shows that most treated segments demonstrated substantial venous obstruction before MT with significant thrombus removal among all patients. Figure 3B shows that effective thrombectomy was achieved among a broad range of DVT severity. These results suggest effective thrombus removal irrespective of baseline thrombus location or burden.

By accepting patients with a contraindication to thrombolytics and not restricting enrollment by symptom duration, outcomes from the CLOUT registry can help to expand the treatable patient population with DVT. In comparison, prior CDT studies excluded patients with subacute and chronic thrombus,^7^, ^8^, ^9^, ^10^, ^11^, ^12^, ^13^, ^14^ and thrombolytic therapies have been shown to be ineffective when treating chronic thrombus.^15^ In CLOUT, more than one-third of treated limbs (35.8%) presented chronic thrombus and more than one-quarter (29.7%) of patients had either a relative or absolute contraindication to thrombolytics. According to the American Society of Chest Physician recommendations,^6^ CDT is contraindicated in patients with a prior intracranial hemorrhage, known structural cerebral vascular lesion or malignant intracranial neoplasm, ischemic stroke or significant head trauma within 3 months, suspected aortic dissection, or active bleeding or bleeding diathesis. There were no major bleeding events in the CLOUT study. In contrast, major bleeding rates in the ATTRACT and CAVA trials were 1.7% at 10 days^10^ and 5% at a median of 5.5 days,^13^ respectively. Outcomes from the CLOUT registry show that MT with the study device can be an effective treatment strategy for this patient population and can provide immediate symptom relief.

The study device shows a favorable safety profile. Acute rates of SAEs, rethrombosis of the targe vessel segment, and mortality were 2.6%, 1.4%, and 0.6%, respectively. Although there is variation in study population and timepoints, we compared these discharge data with the most acute rate of recurrent venous thromboembolism from previously published studies for illustrative purposes. The rate of acute rethrombosis was less than that of patients treated with anticoagulation alone (3.2%-3.6%, during treatment window)^25^ and is comparable with reports from the ATTRACT trial (2.0% within 10 days).^10^ The proven efficacy and lessened risks of MT appear favorable and provide a reasonable rationale for intervention. The entire CLOUT study suggests that further study with MT may provide a greater risk to benefit ratio in the treatment of iliofemoral DVT than seen in prior randomized trials.

The mechanical nature of the study device, along with the absence of thrombolytics, facilitated the rapid removal of thrombus, often in single session (99.4%), with negligible blood loss (median, 40.0 mL), and with most patients (97.8%) requiring no ICU stay. In contrast, thrombolytic-based interventions require attentive monitoring as standard of care^26^ due to bleeding risks and may require multiple sessions.^8^^,^^10^^,^^13^^,^^27^ Also, stents were placed at a comparable rate with the CAVA trial^13^^,^^14^ and at a higher rate than in the ATTRACT trial.^10^

Patient-centered outcomes, including pain and edema, showed significant, immediate improvement through discharge. Outcomes presented in the current study validate the initial findings reported by Dexter et al,^19^ in which the authors also showed sustained reduction of edema and pain to 6 months. In the ATTRACT trial,^10^ patients treated with anticoagulation alone saw reduced pain but increased swelling in the treated limb at 10 days.^10^ Patients treated with pharmacomechanical thrombectomy showed decreased pain and edema at 10 days, although neither were significant when compared with the control group.^10^

In-hospital outcomes from this study are congruent with other publications.^28^^,^^29^ Jolly et al^28^ reported a retrospective single-center review of 96 patients with acute and subacute iliofemoral DVT treated with the same study device. Their experience saw complete or near complete thrombus removal in 97% of treated limbs with no mortality or major bleeding. Similarly, Weissler et al^29^ published a retrospective series of 18 patients treated with the same study device for extensive and provoked iliocaval and iliofemoral DVT, including 3 patients (17%) who had failed prior CDT for the current DVT. Venous flow was reestablished in all patients with no mortality or bleeding complications. These findings, across multiple independent studies, further suggest that the study device is safe and effective in a large patient population with a variety of DVT characteristics.

The CLOUT registry was an observational, single-arm investigation with several limitations. The study framework allowed for physician preference, thus introducing variability related to technical application of the study device, procedural workflow, application of adjunctive therapy, and postthrombectomy anticoagulation regimen. Although stenting is an important part of DVT management and was applied per physician discretion, stent model, sizing, and placement were not recorded. Edema metrics are semiqualitative and can fluctuate in patients relative to body mass index, fluid management, compliance to compression, and interrater variability, which confound clinical significance. In addition, while promising, these results represent immediate in-hospital outcomes, and long-term analysis will direct subsequent study design, guiding future iterations of DVT care and management.

## Conclusion

After complete enrollment, this analysis of the CLOUT registry shows that MT with the ClotTriever System can effectively remove thrombus and has a promising safety profile. Clinically meaningful improvements in edema and pain were observed. These initial results will be confirmed with ongoing follow-up to 2 years and provide rationale for future randomized investigations.
